# Microbial biopriming and germination cooperatively remodel brown lentil seeds (*Lens culinaris* L.) metabolome and antidiabetic functionality

**DOI:** 10.1038/s41538-026-00824-5

**Published:** 2026-04-13

**Authors:** Dina S. Ghallab, Doaa A. Ghareeb, Ehab E. Shawer, Amal A. Othman, Mariam M. Elattar

**Affiliations:** 1 Department of Pharmacognosy, Faculty of Pharmacy, Alexandria University, Alexandria, Egypt; 2 Bio-screening and Preclinical Trial Lab, Biochemistry Department, Faculty of Science, Alexandria University, Alexandria, Egypt; 3 Center of Excellence for Drug Preclinical Studies (CE-DPS), Pharmaceutical and Fermentation Industry Development Center, City of Scientific Research and Technological Applications (SRTA-City), New Burg El-Arab City, Alexandria, Egypt; 4 Research Projects unit, Pharos University, Alexandria, Egypt; 5 National Institute of Oceanography and Fisheries (NIOF), Cairo, Egypt

**Keywords:** Biochemistry, Biotechnology, Chemistry, Plant sciences

## Abstract

Lentil seeds are packed with essential nutrients and health-promoting phytochemicals, yet the dual effect of biopriming and germination on their chemical and functional properties is not fully resolved. Thus, the current study investigates the impact of bacterial biopriming (*B. pomilus* and *E. hormaechei)* followed by germination on reshaping the chemical and biological attributes of lentil seeds using UPLC-MS metabolomics and chemometrics approaches. Sixty-nine metabolites spanning amino acids, phenolic acids, flavonoids, proanthocyanidins, gibberellins, and fatty acids were annotated. OPLS–DA analysis exhibited an obvious separation pattern among the samples reflecting their chemical heterogeneity, where the compounds mainly gallocatechin, phloretin, α-tocotrienol, and eicosapentanoic acid were significantly augmented in sprouted (unprimed) samples, while the sprouted samples bioprimed with *B. pomilus* witnessed a striking accumulation of carboxyvanillic acid, resveratrol, isorhamnetin, gibberellic acid, and gibberellin A4. Further, *E. hormaechei* biopriming significantly fortified rosmarinic acid, syringic acid, quercetin, apigenin, and linolenic acid. Functionally, bioprimed sprouts displayed superior *α*-amylase and *α*-glucosidase inhibition and enhanced glucose uptake relative to raw and unprimed germinated seeds, aligning with higher levels of sinapic acid, apigenin, isorhamnetin, and linolenic acid. These findings position microbe-assisted germination as a sustainable strategy to fortify the functional attributes of lentil seeds for nutraceutical applications.

## Introduction

Diabetes is a rapidly escalating global endocrine disorder, posing significant health and socioeconomic burdens worldwide. It is predicted to affect approximately 10.2% of the global population (578 million) by 2030, rising to 10.9% (700 million) by 2045^1^. The disease is primarily characterized by persistent hyperglycemia resulting from impaired insulin secretion, insulin resistance, or a combination of both, leading to disruptions in glucose uptake, storage, and metabolism^2^. Type 2 diabetes, the most prevalent form, is associated with inadequate insulin production or reduced sensitivity of target tissues to insulin action^3^. If left untreated, chronic hyperglycemia can pose extensive damage to both vascular and nervous systems, leading to serious complications such as kidney failure, nerve damage, limb amputation, vision loss, and an elevated risk of myocardial infarction and stroke^3,4^. In this perspective, to mitigate postprandial glucose spikes, pharmacological agents such as acarbose and miglitol are commonly employed. These drugs act by inhibiting carbohydrate-hydrolyzing enzymes like *α*-glucosidase and *α*-amylase, thereby delaying carbohydrate digestion and slowing glucose absorption in the small intestine^3^. Another therapeutic approach involves agents such as metformin and pioglitazone, which enhance cellular glucose uptake and improve insulin sensitivity, contributing to better glycemic control. However, the use of these medications is often associated with adverse effects, including gastrointestinal discomfort, nausea, weight gain, and fluid retention^5,6^. Consequently, there is a growing interest in exploring natural sources for safer and more sustainable antidiabetic agents.

For centuries, the Fabaceae family stands out among plant families recognized for their notable antidiabetic potential^4,7^. Among which, Lentils (*Lens culinaris* L.), like other pulses, are recognized for their high nutritional density and associated health benefits. Numerus studies highlighted their potential to go beyond basic nutritional support, contributing to the prevention of various chronic conditions, including diabetes, cardiovascular diseases, cancer, aging, and neurodegenerative disorders^8,9^. In addition to serving as a valuable source of plant-based protein, lentils are rich in dietary fiber, essential minerals, and a diverse array of phytochemicals such as phenolic acids, flavanols, saponins, and condensed tannins. These diverse bioactive compounds contributed significantly to lentil beneficial health functionalities^10,11^.

Lentils have demonstrated significant antidiabetic properties, supported by both in vitro and in vivo studies. Consumption of cooked lentils was associated with reduced postprandial blood glucose and insulin response, particularly in individuals with type 2 diabetes^9^. These effects were attributed to lentils’ unique starch profile—comprising both rapidly digestible and resistant starch as well as their high concentrations of protein, dietary fiber, and phenolic compounds^12,13^. Lentils also exhibited potent inhibitory activity against *α*-glucosidase and *α*-amylase enzymes, critical to carbohydrate digestion and glucose absorption^14^.

Considering the fast-paced field of nutritional therapy, different methods have been introduced for improving the nutritional and functional qualities of edible seeds, surpassing the gut digestibility of balanced macro and micronutrients, and limiting the bioavailability of anti-nutrients^15^.

Germination, also termed as sprouting, is one of the fortunate green modification strategies that is widely being adopted on seeds to significantly boost their bioactive compounds and improve nutrient bioavailability, transforming ordinary seeds into valuable functional ingredients for health and disease prevention^16^. Seed priming is a rather green modification technology implicated in improved germination, periodic adjustments enhancement, metabolic reorganization, and overall crop growth or yield^17^. The terms “seed priming” and “crop enhancement”—often used interchangeably—refer to physical seed enhancement techniques that improve performance even under sub-optimal conditions^17^. These techniques employ various priming (bio-stimulating) agents, such as plant growth-promoting microorganisms (PGPMs), including beneficial bacteria, fungi, and algal extracts^18^.

Seed coating with beneficial microorganisms enhances agricultural performance under fluctuating climatic conditions by promoting enzymatic activity, facilitating biochemical cycling and nutrient translocation, and strengthening plant defense mechanisms against various stresses^19^. Beyond improving nutrient availability and stress tolerance, biopriming agents also favorably stimulate the biosynthesis and restoration of diverse bioactive metabolites and phytohormones, while increasing water and nutrient uptake under limited soil moisture conditions^20^.

Correspondingly, seed priming with microbial inoculants has gained considerable attention as a promising strategy not only for improving overall field performance but also for regulating and accumulating secondary metabolites of nutritional and therapeutic relevance. A review of previous studies highlights the positive impact of plant growth-promoting microorganisms (PGPMs) on seed germination and metabolic regulation, ultimately leading to increased accumulation of bioactive compounds and associated beneficial traits. For instance, inoculation of *Chryseobacterium balustinum* Aur9 in soybeans has been shown to enhance flavonoid efflux and strengthen plant immunity against *Sclerospora graminicola* infection^21^. Similarly, combined application of *Pseudomonas* sp. and *Azospirillum* sp. in *Zea mays* has been reported to alter secondary metabolite profiles, particularly benzoxazinoids and diethyl phthalate^22^. Furthermore, PGPMs can influence phytohormone productivity, playing multifaceted roles in plant growth and development^23^. Specifically, species such as *Azospirillum* spp., *Azotobacter* spp., and *Bacillus* spp. have been documented to induce gibberellin and cytokinin production, thereby modifying root architecture, stimulating cell division, and promoting meristematic tissue differentiation in various plant species^24^.

Despite significant progress, certain aspects remain vague, particularly the understanding of metabolic patterns and their chemical distribution following seed germination and biopriming with different beneficial stimulants. Addressing these gaps is essential for developing rational strategies to enhance the production of bioactive compounds of health significance in an economical manner.

To this end, the current study employed an ultra-high-performance liquid chromatography–mass spectrometry (UPLC–MS/MS) metabolomics approach combined with chemometric analysis to capture the dynamic spectrum of metabolites in lentil seeds primed with different beneficial bacteria during germination. Furthermore, to experimentally validate the impact of biopriming and germination on the enrichment of valuable compounds, bioprimed seeds were screened in vitro for glucose uptake enhancement and *α*-amylase and *α*-glucosidase inhibitory activities. In parallel, OPLS-guided modeling was used to elucidate the metabolic fingerprints underlying these bioactivities.

To date, no previous study has integrated microbe-assisted germination with UPLC–MS/MS metabolomics and OPLS-based modeling to connect strain-dependent metabolite reprogramming with antidiabetic functionality in lentil sprouts. To our knowledge, this dual interrogation, simultaneously profiling chemical remodeling under two beneficial bacterial primings and quantifying its link to enzyme inhibition and glucose uptake in a single framework, provides the first evidence supporting biopriming as an environmentally sustainable strategy for developing legume‑derived nutraceuticals. These findings highlight the potential of microbial priming to enhance the functional attributes of sprouted legumes and advance nutraceutical and functional food applications globally.

## Results and discussion

### Characterization of the main metabolites detected in lentil samples using UPLC-QqQ-MS/MS

**Fig. 1:**
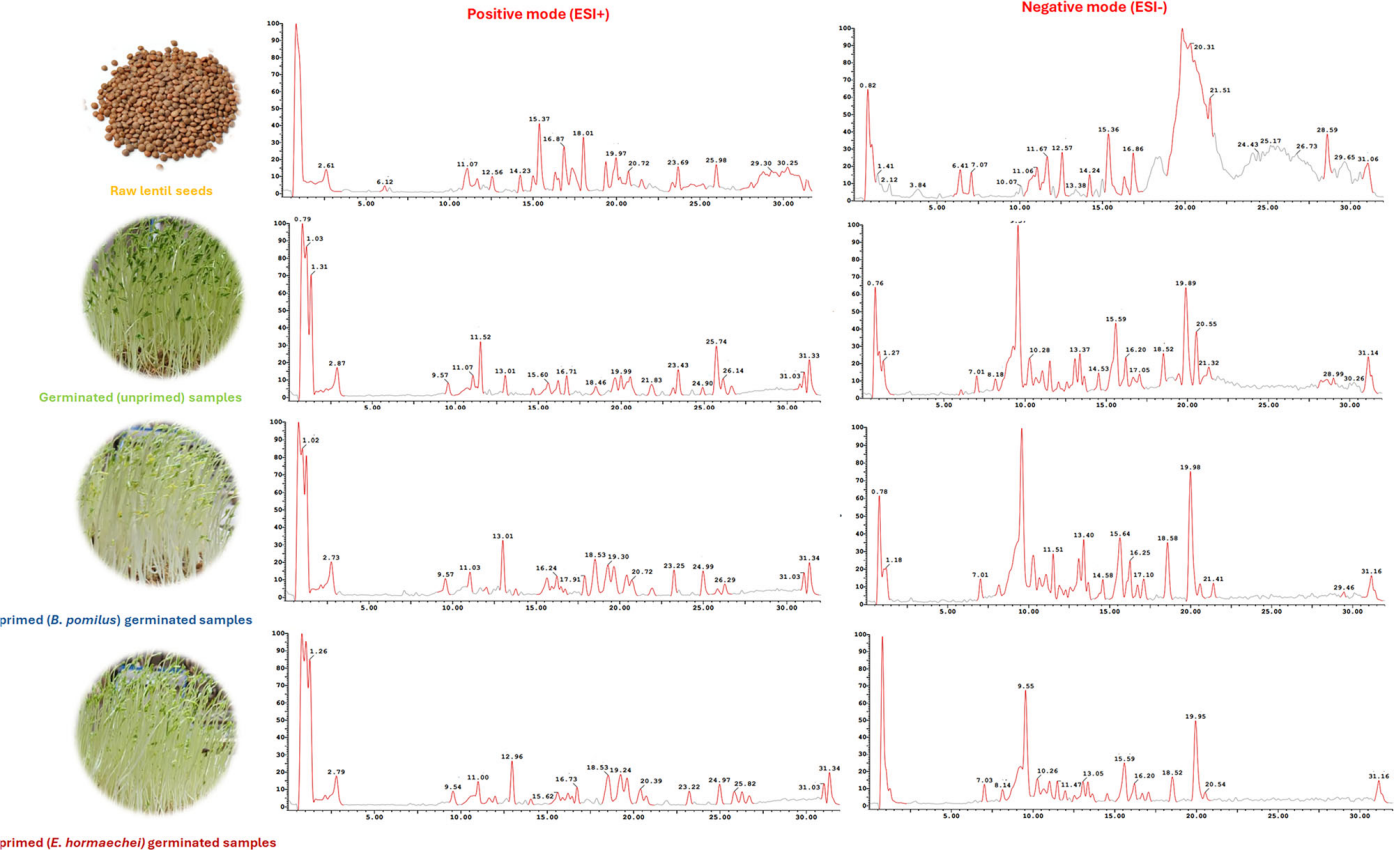
Base peak chromatograms (BPCs) of different lentil samples in both positive and negative polarity modes.

In the present study, a UPLC-QqQ-MS/MS-based metabolomics approach was utilized to comprehensively assess the impact of germination and bacterial biopriming on the chemical profile of lentil (*Lens culinaris* L.) seeds. Four sample types were analyzed: raw seeds, germinated seeds, and seeds bioprimed with *Enterobacter hormaechei* and *Bacillus pumilus*. A total of 69 chromatographic peaks were annotated, encompassing a wide range of metabolites including sugars, amino acids and peptides, flavonoids, proanthocyanidins, phenolic acids, sapogenins, gibberellins, fatty acids, phospholipids, and phytosterols. Compounds identification was achieved through retention time alignment, characteristic fragmentation patterns, reference standards, and corroborating literature. Base peak chromatograms (BPCs) acquired in both positive and negative ionization modes are presented in Fig. 1. Additionally, Table 1 summarizes the annotated compounds, including their mass spectral data, retention times, molecular formulas, and chemical classifications. Subsequent sections delineate the approach adopted for annotating the major chemical classes. Analytical precision and reproducibility were validated using quality control samples.

**Table 1:** Metabolites identified in lentil samples by UPLC-ESI-MS/MS under both positive and negative ionization modes

No.	RT (min)	Compound name	Ion type	Chemical class	Mwt	Formula	Ms^n^ Fragments	Reference
**1**	0.47	Dulcitol	M+Na	Sugar alcohol	182.17	C₆H₁₄O₆	187, 169, 151	^77^
**2**	0.55	Trehalose	M-H	Disaccharide	342.29	C₁₂H₂₂O₁₁	179, 161, 89	^78^
**3**	0.63	Allantoic acid	M-H	N-carbamoyl-*α*-amino acid (Urea)	176.13	C₄H₈N₄O₄	156, 138, 77	^79^
**4**	0.7	Choline	M^+^	Quaternary ammonium salt	104.17	C₅H₁₄NO	86.2, 45.3	^80^
**5**	0.83	Cysteine	M + H	Amino acid	121.2	C₃H₇NO₂S	105, 104, 88, 76, 59	^31^
**6**	0.9	Citrulline	M + NH_4_	Amino acid	175.2	C₆H₁₃N₃O₃	113, 115, 158	^81^
**7**	1.09	Glutamyl-valine	M + H	Dipeptide	246.3	C₁₀H₁₈N₂O₅	229, 130.2, 118.12, 102.1, 72	^82^
**8**	1.19	Succinic acid	M-H	Organic acid	118.1	C₄H₆O₄	99.1, 73	^24^
**9**	1.23	Lysine	M + H	Amino acid	146.2	C₆H₁₄N₂O₂	130, 84, 56	^31^
**10**	1.34	Saccharopine	M + H	Amino acid	276.28	C₁₁H₂₀N₂O₆	213, 130, 84	^31^
**11**	1.45	Leucine	M + H	Amino acid	131.2	C₆H₁₃NO₂	86, 44, 43	^31^
**12**	1.52	Methylcitrate	M-H	Organic acid derivative	206.15	C₇H₁₀O₇	87, 101, 125	^83^
**13**	1.62	Threonine	M + NH_4_	Amino acid	119.11	C₄H₉NO₃	102, 74, 56	^31^
**14**	1.9	Adenosine	M + H	Nucleoside	267.24	C₁₀H₁₃N₅O₄	136, 151.12, 119.3	^84^
**15**	2.08	Epigallocatechin	M-H	Flavanol	306.26	C₁₅H₁₄O₇	139, 123, 107	^85^
**16**	2.17	Valyl- Phenylalanine	M + H	Dipeptide	264.32	C₁₄H₂₀N₂O₃	247, 166, 100	^86,87^
**17**	2.27	Tryptophan	M + H	Amino acid		C₁₁H₁₂N₂O₂	188, 146, 118	^31^
**18**	2.69	Hydroxybenzoic acid	M-H + HCOO	Phenolic acid	138.12	C₇H₆O₃	119, 93, 75	^88^
**19**	2.76	Phenylalanine	M + H	Amino acid	165.18	C₉H₁₁NO₂	148, 122, 120, 103	^31^
**20**	2.87	Sinapic acid	M + H	Phenolic acid	224.21	C₁₁H₁₂O₅	207, 181, 165	^24^
**21**	3.81	Vanillic acid-4-*O*-glucoside	M + H	Phenolic acid	330.28	C₁₄H₁₈O₉	169, 151, 137	^38^
**22**	4.09	Dihydroxybenzoic acid-*O*-dipentoside	M + H + HCOO	Phenolic acid	418.34	C₁₇H₂₂O₁₂	287, 155, 137, 109	^89^
**23**	4.69	Protocatechuic acid	M + H + HCOO	Phenolic acid	154.12	C₇H₆O₄	137, 109	^90^
**24**	5.03	Caffeic acid	M-H	Phenolic acid	180.15	C₉H₈O₄	163, 137	^91^
**25**	5.32	Rosmarinic acid	M-H	Phenolic acid	360.3148	C₁₈H₁₆O₈	145, 135, 117	^92^
**26**	6.03	Chlorogenic acid	M + H	Phenolic acid	354.31	C₁₆H₁₈O₉	241, 121	^93^
**27**	6.13	Carboxyvanillic acid	M + H	Phenolic acid	212.16	C₉H₈O₆	185, 169	^89^
**28**	6.22	Syringic acid	M + H + HCOO	Phenolic acid	198.17	C₉H₁₀O₅	140, 125	^91^
**29**	6.29	Vanillic acid	M + H	Phenolic acid	168.15	C₈H₈O₄	154.14, 151.2, 137, 125	^24^
**30**	6.41	Ferulic acid	M + H	Phenolic acid	194.18	C₁₀H₁₀O₄	180, 177, 163.2	^94^
**31**	7.03	Quercetin	M-H	Flavonol	302.03	C₁₅H₁₀O₇	271, 179, 151	^82^
**32**	7.11	Protocatechuic aldehyde	M + H	Hydroxybenzaldehyde	138.12	C₇H₆O₃	121, 111, 110	^95^
**33**	7.56	5,7-dihydroxy flavone	M + H	Flavone	254.29	C₁₅H₁₂O₄	253, 237, 151	^96^
**34**	7.79	Apigenin	M + H	Flavone	270.23	C₁₅H₁₀O₅	253, 241, 153	^97^
**35**	8.7	Resveratrol	M + H	Stilbenoid	228.24	C₁₄H₁₂O₃	211, 185, 135	^39^
**36**	9.4	Trihydroxy-megastigmadien-one hexoside	M-H	Megastigmane glycoside	402.19	C₁₉H₃₀O₉	239, 221, 203.12, 185.3	^98^
**37**	9.55	Naringenin	M+Na	Flavanone	272.25	C₁₅H₁₂O₅	152, 120, 108	^24^
**38**	9.65	12-Oxo-Dodecenoic Acid	M + H	Fatty acid	212.28	C₁₂H₂₀O₃	195, 185, 177	^99^
**39**	10.05	Dimethyl quercetin	M-H	Flavonol	330.2889	C₁₇H₁₄O₇	285, 255, 151, 137	^100^
**40**	10.27	N-Feruloyl serotonin	M + H	N-acyl serotonin	352.4	C₂₀H₂₀N₂O₄	335, 195, 160	^101^
**41**	12.5	Procyanidin A2	M + H	Proanthocyanidin	576.5	C₃₀H₂₄O₁₂	451, 291, 289, 153, 137	^36^
**42**	11.51	Phloretin	M + H	Dihydrochalcone	274.26	C₁₅H₁₄O₅	257.2, 239.13, 153, 123	^33^
**43**	12.95	Isorhamnetin	M + H	Flavonol	316.26	C₁₆H₁₂O₇	302, 229, 153	^24^
**44**	15.27	Soyasapogenol B	M + H	Triterpenoid sapogenin	458.72	C₃₀H₅₀O₃	441.4, 423.4, 405.4, 219, 207	^40^
**45**	15.37	LysoPC 16:0	M + H	Lysophosphatidylcholine	495.63	C₂₄H₅₀NO₇P	258, 240, 184	^102^
**46**	16.24	Hydroxy linolenic acid	M + H	Fatty acid	294.429	C₁₈H₃₀O₃	133, 119, 81	^99^
**47**	16.47	Gibberellin A_1_	M + H	Diterpenoid acid	348.39	C₁₉H₂₄O₆	331, 313, 285	
**48**	16.76	LysoPC 15:1	M + H	Lysophosphatidylcholine	479.6	C₂₃H₄₆NO₇P	258, 240, 184	^102^
**49**	17.22	Linolenic acid	M + H	Fatty acid	278.43	C₁₈H₃₀O₂	261, 243, 235, 233, 219, 56	^24^
**50**	18.29	Linolenoyl glycerol	M + H	Monoacylglycerol (Monoglyceride)	352.5	C₂₁H₃₆O₄	335.3, 279.2, 93	^99^
**51**	18.4	Gibberellin A_4_	M + H	Diterpenoid acid	332.39	C₁₉H₂₄O₅	315, 287, 269	
**52**	18.51	2-Hydroxy palmitic acid	M-H	Fatty acid	272.42	C₁₆H₃₂O₃	253, 227, 73	^99^
**53**	19.41	Linoleoyl glycerol	M + H	Monoacylglycerol (Monoglyceride)	354.53	C₂₁H₃₈O₄	337.2, 281, 93	^99^
**54**	19.87	Soyasapogenol C	M + H	Triterpenoid sapogenin	440.70	C₃₀H₄₈O₂	423.3, 405.3, 207.12	^40^
**55**	20.37	*α*-Tocotrienol	M+Na	Vitamin E analog	424.65	C₂₉H₄₄O₂	410.5, 407, 205, 165	^103^
**56**	20.39	Linoleic acid	M + H	Fatty acid	280.44	C₁₈H₃₂O₂	263, 237, 171.2, 155	^99^
**57**	20.47	Gibberellin A_3_ (Gibberellic acid)	M + H	Diterpenoid acid	346.36	C₁₉H₂₂O₆	329, 311, 301, 285	^99^
**58**	20.66	Oleoyl Glycerol	M + H	Monoacylglycerol (Monoglyceride)	356.54	C₂₁H₄₀O₄	339.2, 283, 93, 75	^99^
**59**	21.78	Ricinoleic acid	M+Na	Fatty acid	298.46	C₁₈H₃₄O₃	281, 185, 99	^99^
**60**	21.96	Eicosapentanoic acid	M+Na	Fatty acid	302.45	C₂₀H₃₀O₂	259, 171, 131	^99^
**61**	23.16	Oleic acid	M + H	Fatty acid	282.46	C₁₈H₃₄O₂	265, 239, 171.12, 113.4	^24^
**62**	23.32	Methyl linolenic acid	M+Na	Fatty acid	292.46	C₁₉H₃₂O₂	147, 119, 93	^24^
**63**	23.34	Campestrol	M + H	Phytosterol	400.68	C₂₈H₄₈O	383, 315, 273, 255, 213	^99^
**64**	23.58	Hydroxy octadecanoic acid	M+Na	Fatty acid	300.47	C₁₈H₃₆O₃	213, 199, 183	^99^
**65**	24.48	Stearic acid	M+Na	Fatty acid	284.48	C₁₈H₃₆O₂	267, 241, 239.4	^24^
**66**	26.43	Eicosadienoic acid	M + H	Fatty acid	308.49	C₂₀H₃₆O₂	249, 205, 179	^104^
**67**	28.49	Stigmasterol	M + H	Phytosterol	412.7	C₂₉H₄₈O	395, 297.2, 273, 255, 213	^84^
**68**	28.63	Arachidic acid	M+Na	Fatty acid	312.53	C₂₀H₄₀O₂	295, 269, 255	^99^
**69**	29.59	Erucic acid	M + H	Fatty acid	338.57	C₂₂H₄₂O₂	321, 295, 281	^104^

^*^RT Retention time, *Mwt* molecular weight.

### Sugars and organic acids

In this study, two distinct compounds belonging to the sugar class were identified. The first, compound 1, was characterized as a sugar alcohol, while the second, compound 2, was classified as a disaccharide. Compound 2 gave a deprotonated parent ion at m/z 341.49. Fragmentation of the precursor ion yielded diagnostic ions at m/z 179, corresponding to a deprotonated glucopyranose unit [C_6_H_11_O_6_]^−^ resulting from glycosidic bond cleavage; m/z 161, formed by the loss of a water molecule from the glucopyranose unit [C_6_H_9_O_5_]−; and m/z 89, a smaller fragment likely arising from cross-ring cleavage within the hexose structure, assigned to [C_3_H_5_O_3_]−. Hence, Compound **2** was annotated as trehalose^12^ and further confirmed by a reference standard. Similarly, compound 1 gave a quasi-molecular ion at m/z 205.2 in the positive ion mode, likely a sodium adduct [M+Na] ^+^. Upon tandem MS/MS analysis, this ion underwent sequential neutral losses of water molecules. The major fragment ions were detected at m/z 187, 169, and 151, corresponding to the loss of one to three water molecules, respectively, which reflected the molecule’s symmetrical structure and high hydroxyl content. Accordingly, Compound 1 was assigned as dulcitol^25^.

Further, two organic acid derivatives were assigned in the current analysis (compounds 8 and 12). Compound 8 showed its deprotonated molecular ion at an m/z of 117.17 (Table 1). MS/MS spectrum of the compound was dominated by an intense fragment at m/z 73, generated by the neutral loss of carbon dioxide (CO₂), and a secondary fragment observed at m/z 99, resulting from the loss of water (H₂O). Consequently, the compound was annotated as succinic acid in line with previous literature^26^.

### Amino acids, dipeptides and basic compounds

Lentils are an excellent plant-based protein source, comprising 20–30% protein with a favorable amino acid profile, containing almost all essential amino acids^27^. In the current analysis, this compound class was represented by peaks 3, 4, 5, 6, 7, 9, 10, 11, 13, 14, 16, 17, 19, and 40, and constituted the most prevalent group among those identified in the lentil samples. Compound **7** exhibited a dominant protonated molecular ion [M + H]^+^ at m/z 247.28. Tandem MS analysis revealed characteristic peptide fragmentation, primarily through cleavage of the amide bond. This yielded two major sequence-defining fragment ions at m/z 130.2 and 118.12 corresponding to the b₁ ion of glutamic acid residue, and the y₁ ion of valine, respectively. Additional structural confirmation was provided by diagnostic immonium ions, notably observed at m/z 72 for valine and at m/z 102.1 for glutamic acid, together with another prominent ion detected at m/z 229, indicating a neutral loss of water from the precursor ion. Collectively, the observed ions provided a robust fragmentation signature for the identification of the compound as the dipeptide Glutamyl valine^28,29^. Similarly, Compound 4 gave a prominent precursor ion at m/z 104.17 [M + H]^+^. Subsequent MS/MS analysis revealed a major fragment ion at m/z 45 consistent with the loss of a neutral trimethylamine group (N(CH_3_)_3_), and another one at m/z 86 corresponding to the loss of a water molecule (Table 1). These fragmentation features strongly indicated the identity of the compound as choline^30^, which was further confirmed by a reference standard. The peak of compound 5 was detected at m/z 122.21 [M + H]^+^ and subsequently identified as cysteine. Tandem MS analysis revealed a series of key fragment ions at m/z 104, 76, and 59 corresponding to the sequential losses of water, carbon monoxide, and NH_3_, respectively. Additionally, other notable fragments included m/z 105, resulting from ammonia loss, and m/z 88, associated with the elimination of hydrogen sulfide. The presence of these specific ions enabled the annotation of the compound as cysteine^29,31^.

Compound **19** exhibited a quasi-molecular ion at *m/z* 166.31 [M + H] ⁺, showing a fragmentation pattern consistent with phenylalanine. The spectrum was dominated by an intense base peak at m/z 120, consonant with the benzyl-immonium ion (C₈H₁₀N⁺), a highly characteristic fragment for a phenylalanine residue. Further supporting this assignment were significant fragment ions observed at m/z 148 and 122, indicating neutral losses of water and CO_2_, along with an additional fragment at m/z 103, corresponding to a tropylium-like cation (C₈H₇⁺)^29,31^. In the same way, the molecular ion peak of compound 14 was detected at m/z 268.34 [M + H]^+^. The observed MS/MS spectrum revealed a dominant peak at m/z 136 attributed to protonated adenine (C₅H₆N₅⁺), upon cleavage of the N-glycosidic bond—a highly diagnostic feature of purine nucleosides. A complementary fragment at m/z 151.12, corresponding to the protonated ribose moiety (C₅H₁₁O₅⁺), together with another fragment at m/z 119.3, indicating the neutral loss of NH_3_ (17 Da) from the adenine base, reinforced the structural assignment of the compound as adenosine^32^.

### Flavonoids and proanthocyanidins

The flavonoids identified in the analyzed lentil samples encompassed several subclasses, including flavanols (compound 15), flavones (compounds 33 and 34), flavan-3-ols (compounds 31, 39, and 43), flavanones (compound 37), dihydrochalcones (compound 42), together with one A-type proanthocyanidin dimer (dimeric flavan-3-ol), represented by compound 41. Compound 42 exhibited a protonated molecular ion at m/z 275.53. Tandem MS analysis revealed a fragmentation pattern characteristic of dihydrochalcones. Two key ions were observed at m/z 257.2 and 239.13, consistent with sequential losses of two molecules of water, denoting the presence of multiple hydroxyl groups (Table 1). Additionally, two highly diagnostic fragments were observed at m/z 153 and 123 corresponding to the A-ring and B-ring fragments resulting from retro-Diels–Alder (RDA) cleavage of ring C, supporting the annotation of the compound as phloretin in alignment with previous reports^33^.

Compound 31 presented a deprotonated molecular ion at m/z 301.35. Fragmentation spectrum revealed a series of diagnostic product ions at m/z 283 and 273 corresponding to neutral losses of water and carbon monoxide, respectively. Further, two highly informative fragment ions were noted at m/z 151 and 179 corresponding to [^1,3^A] ^–^ and [^1,2^A] ^–^ ions, resulting from RDA cleavage of the C-ring. Together, these fragmentation characteristics provided compelling evidence for the structural identity as quercetin, which was further confirmed by a reference standard^34,35^. In a similar manner, compound 41 exhibited a predominant [M + H] ⁺ ion at m/z 577.45, alongside a doubly charged species at m/z 289 ([M + 2H] ²⁺), indicative of a dimeric nature. Subsequent MS/MS analysis of the precursor ion yielded a characteristic ion pair at m/z 291and 289, corresponding to the protonated epicatechin monomer units generated via quinone methide cleavage of the interflavan bond. Crucially, the spectrum was dominated by a product ion at m/z 451.1, corresponding to a neutral loss of a phloroglucinol moiety (126 Da), which is a hallmark of the additional C2 → O7 ether bond characteristic of A-type proanthocyanidin dimers. The structural assignment was further substantiated by the presence of ions at m/z 153 and 137, arising from RDA fission of the flavan-3-ol subunits. On the basis of its unique fragmentation profile, the compound was conclusively identified as Procyanidin A2^36,37^.

### Phenolic acids and other phenolic compounds

In the current investigation, twelve phenolic acids were identified, corresponding to compounds 18, 20, 21, 22, 23, 24, 26, 27, 28, 29, 30, and 32, along with one stilbenoid represented by compound 35. Compound **29** showed a protonated precursor ion at m/z 169.39. Fragmentation of the parent ion revealed a series of characteristic fragment ions, including ions at m/z 154.14, indicating loss of a methyl radical, and at m/z 151.2 corresponding to water loss. Other diagnostic ions were noticed at m/z 137 and 125, consistent with neutral losses of methanol and CO₂, respectively, suggesting the assignation of the compound as vanillic acid, which was further confirmed by a reference standard^26^. Similarly, the precursor ion of compound 21 was detected at m/z 331.49. MS/MS analysis revealed a fragment ion at m/z 169—consistent with vanillic acid upon elimination of a glucosyl moiety (162 Da), suggesting that compound 21 is a glycosylated derivative of vanillic acid. Additional diagnostic fragments were observed at *m/z* 151&137 which further supported the annotation of compound 21 as vanillic acid-4-*O*-glucoside^38^. In the same respect, compound 30 gave a protonated molecular ion at m/z 195.38. Fragmentation of the precursor ion generated a key fragment ion at m/z 180, consonant with the loss of a methyl radical (15 Da), together with other diagnostic ions at m/z 177 attributed to neutral loss of a water molecule (18 Da), and at m/z 163.2, corresponding to the loss of methanol (32 Da) (Table 1), which supported the presence of a methoxylated phenolic structure. Hence, the compound was annotated as ferulic acid^26^.

Compound 35 gave its precursor ion at m/z of 229.38 [M + H]^+^. Subsequent collision-induced dissociation (CID) of the parent ion yielded a fragmentation pattern characteristic of the stilbene core structure. Prominent fragment ions were observed at m/z 211 and 185, resulting from the neutral loss of water (18 Da) and C₂H₄O (44 Da), respectively. A particularly intense and key fragment was identified at m/z 139, corresponding to a significant rearrangement and cleavage of the molecular backbone, indicative of the stilbene core structure. The observed mass data provided definitive evidence for the identification of the compound as resveratrol^39^.

### Terpenoids and phytosterols

Two triterpenoid sapogenins were annotated in the current study, represented by compounds 44 & 54. Compound 54 exhibited a protonated molecular ion at m/z 441.52. Subsequent CID of the parent ion yielded prominent fragment ions at m/z 423.3 and m/z 405.3, indicative of sequential losses of one and two molecules of water. Furthermore, a characteristic triterpenoid cleavage fragment was detected at m/z 207.12 (Table 1). Comparison of these fragmentation patterns with reference data and databases confirmed the identity of the compound as Soyasapogenol C^40^. Similarly, compound 44 showed a protonated molecular ion at m/z 459.51. Its fragmentation spectrum revealed sequential losses of water, evidenced by fragment ions at m/z 441.4, 423.4, and 405.4, corresponding to the loss of one, two, and three water molecules, respectively. The presence of triterpenoid-specific fragments, at m/z 207 and 219, further supported its structural assignment. Based on these observations, particularly the higher molecular weight and the additional water loss compared to Soyasapogenol C, compound 44 was unambiguously annotated as Soyasapogenol B^34^.

Further, two phytosterols were detected in our study. Based on the mass measurements and detailed fragmentation patterns, these compounds were subsequently identified and annotated as campesterol (compound 63) and stigmasterol (compounds 67)^35,41^. Both compounds exhibited protonated molecular ions [M + H] ^+^, with campesterol (C₂₈H₄₈O) detected at m/z 401.66 and stigmasterol (C₂₉H₄₈O) at m/z 413.64 (Table 1). The fragmentation spectra of both compounds revealed several common product ions, characteristic of the phytosterol class. Notably, a prominent fragment at m/z 383 for campesterol and *m/z* 395 for stigmasterol corresponded to the neutral loss of a water molecule from the hydroxyl group at the C-3 position. This dehydration step is a hallmark of sterol fragmentation and typically yields the most intense peak in the spectrum. Further fragmentation of the steroidal core produced shared ions at m/z 289, 273, and 255, arising from cleavage within the tetracyclic ring system and partial loss of the side chain, and considered as class-specific markers. Additionally, both compounds exhibited a fragment at m/z 213, attributed to RDA cleavage within the A/B rings of the steroid nucleus, and another one at m/z 257, corresponding to the combined loss of water and the side chain, further supporting the proposed structures. Beyond these shared features, diagnostic ions enabled the differentiation between the two compounds. Campesterol produced a unique fragment at m/z 315, resulting from the cleavage of its saturated side chain. In contrast, stigmasterol generated a distinct fragment at m/z 297.2, which served as a key structural marker for stigmasterol due to the presence of a Δ²² double bond in its side chain^42^.

### Gibberellins

Three gibberellins, 47, 51, and 57, were detected in the current analysis, showing the highest abundance in the bioprimed germinated lentil samples. These compounds were readily identified as Gibberellins A₁ (GA₁), A₄ (GA₄), and A₃ (GA₃, gibberellic acid), respectively (Table 1). These structurally similar diterpenoids were distinguished based on their characteristic neutral losses during CID, particularly the sequential or simultaneous elimination of water (18 Da) and carbon monoxide (28 Da)^43^. The quasi-molecular ions, along with the distinct fragmentation spectra and specific m/z values of the major product ions, provided diagnostic fingerprints unique to each compound. For GA₁, the protonated precursor ion was observed at m/z 349.43. The fragmentation profile was characterized by the sequential loss of two molecules of water, typical for a 13- and 3-hydroxylated structure. Key fragments were detected at *m/z* 331 [M + H − H₂O] ⁺ and 313 [M + H − 2H₂O] ⁺. Further fragmentation yielded a prominent ion at m/z 285, corresponding to the loss of 2H₂O and CO. Regarding GA₃, it produced a protonated ion at m/z 347.31. Its fragmentation spectrum, while similar in neutral losses, was distinct from GA₁ due to the presence of a C-3, C-4 double bond, showing a difference in the abundance of ions. The major fragments observed were at m/z 329 and 311, corresponding to the sequential loss of one and two molecules of water, and a significant ion at m/z 301, resulting from the combined loss of H₂O and CO. Additionally, a characteristic fragment at m/z 285 corresponds to the combined loss of H₂O and CO_2_. In contrast, GA₄, a non-13-hydroxylated gibberellin, yielded a precursor ion at m/z 333.5. Its fragmentation pattern uniquely displayed the neutral loss of only one water molecule. The primary fragment was identified at m/z 315 [M + H − H₂O] ⁺. Subsequent neutral loss of a CO molecule resulted in a fragment at m/z 287, followed by a loss of a second water molecule to form m/z 269. The absence of a C-13 hydroxyl group prevented a second 18 Da water loss until further complex rearrangements occurred, providing a clear marker for its identification^43^.

### Fatty acids and their derivatives

UPLC-MS/MS analysis of the examined lentil samples enabled the characterization of 12 fatty acids (peaks 38, 46, 49, 52, 56, 59, 60, 61, 62, 64, 68, and 69) and three monoacylglycerols (peaks 50, 53, and 58). The mass spectra of these compounds exhibited both sodiated [M+Na]⁺ and protonated molecular ions. Compound 65 showed its protonated molecular ion [M + H]⁺ at m/z 285.6, along with product ions at m/z 267 and 241 ascribed to neutral losses of H₂O and CO₂, suggesting a C18 fatty acid. Further, a key ion was noticed at m/z 239.4 ([M + H-H₂O-C₂H₄] ⁺), generated via charge-remote cleavage of the saturated hydrocarbon chain. The absence of any fragments indicative of unsaturation, combined with the precursor ion mass, allowed the annotation of the compound as stearic acid (C18:0)^44^, which was further confirmed by a reference standard. Similarly, compound 61 gave a distinct [M + H]⁺ ion at m/z 283.5, indicating the presence of a single double bond. Its MS/MS spectrum also featured the primary water and CO_2_ loss fragments at m/z 265 and 239. Critically, the spectrum contained a pair of highly diagnostic product ions at m/z 171.12 and 113.4 (Table 1). These ions are consistent with allylic cleavage on either side of the C9-C10 double bond and are a signature fragmentation pattern for a Δ^9^ monounsaturated fatty acid. Taken together, the compound was conclusively identified as oleic acid (C18:1). In a similar manner, compound **56** showed a protonated ion at m/z 281.5, and daughter ions at *m/z* 263 and 237, corresponding to a C18 fatty acid with two degrees of unsaturation. The presence of a different set of diagnostic ions at m/z 171.2 and 155, which is highly characteristic of a conjugated Δ^9,12^ diene system, confirmed the identity of the compound as linoleic acid (C18:2)^44^.

Compounds 50, 53, and 58 revealed a fragmentation pathway characteristic of monoacylglycerols (MAGs) involving the neutral loss of water from the glycerol backbone. More diagnostically, cleavage of the ester bond yielded a protonated fatty acid ion ([FA + H] ⁺), which served as a direct identifier of the fatty acyl chain composition. This was supported by the presence of common glycerol backbone fragments, most notably the ion at m/z 93 ([C₃H₉O₃] ⁺)^45^. The peak of compound 58 was detected at m/z 357.58 [M + H]⁺. Its fragmentation spectrum yielded a highly diagnostic ion at m/z 283, corresponding to protonated oleic acid ([18:1 + H]⁺). The presence of the common dehydrated precursor ion at m/z 339.2 and the glycerol backbone fragment at m/z 93 and 75 [Glycerol—H_2_O] provided further confirmation, leading to the annotation of the compound as oleoyl glycerol (MAG 18:1). Similarly, the precursor ion of compound 53 was detected at m/z 355.64 [M + H]⁺. MS/MS analysis produced a key ion at m/z 281, which corresponds to protonated linoleic acid ([18:2 + H]⁺). Based on this unique fatty acyl fragment and the supporting loss of water to form an ion at m/z 337.2, the compound was annotated as linoleoyl glycerol (MAG 18:2). Finally, compound 50 gave its protonated ion at m/z 353.6. The observation of a diagnostic fragment at m/z 279.2, representing protonated *α*-linolenic acid ([18:3 + H]⁺), enabled its identification as linolenoyl glycerol (MAG 18:3).

### Phospholipids

Mass spectral analysis of lentil samples enabled the characterization of two phospholipid species, assigned to the lysophosphatidylcholine (lysoPC) subclass, corresponding to peaks 45 and 48 (Table 1). Compound 45 revealed a prominent precursor ion at m/z 496.74 [M + H]^+^. MS/MS fragmentation of this ion produced a highly abundant and diagnostic product ion at m/z 184, corresponding to the phosphocholine headgroup ([C₅H₁₅NO₄P]⁺), suggesting a lysoPC species. The spectrum also showed a highly diagnostic daughter ion at m/z 240, indicating a major neutral loss of 256 Da, consistent with the removal of a palmitic acid (16:0) moiety. A complementary intense fragment was observed at m/z 258 corresponding to the loss of the 16:0 ketene (238.4 Da) (Table 1), which further confirmed the fatty acyl chain composition. Collectively, this allowed for the annotation of the compound as 1-palmitoyl-sn-glycero-3-phosphocholine (LysoPC 16:0)^46^. Subsequently, compound 48 showed a distinct precursor ion at m/z 480.69. Its MS/MS spectrum also yielded the characteristic phosphocholine ion at m/z 184, confirming it as another Lysopc species. A key neutral loss of 240 Da was observed, corresponding to a pentadecenoic acid (15:1) chain and producing a fragment at m/z 240. The corresponding loss of the 15:1 ketene (222 Da) was also confirmed by the fragment ion noticed at m/z 258.2. Taken together, this compound was assigned as 1-pentadecenoyl-sn-glycero-3-phosphocholine (LysoPC 15:1)^46^.

To sum up, the applied approach enabled a high-confidence annotation of 69 metabolites covering primary and secondary classes. This dataset establishes a comprehensive chemical baseline across raw, germinated, and bioprimed sprouts, enabling downstream discrimination and bioactivity correlation analyses.

### Metabolic trends among the analysed lentil samples

**Fig. 2:**
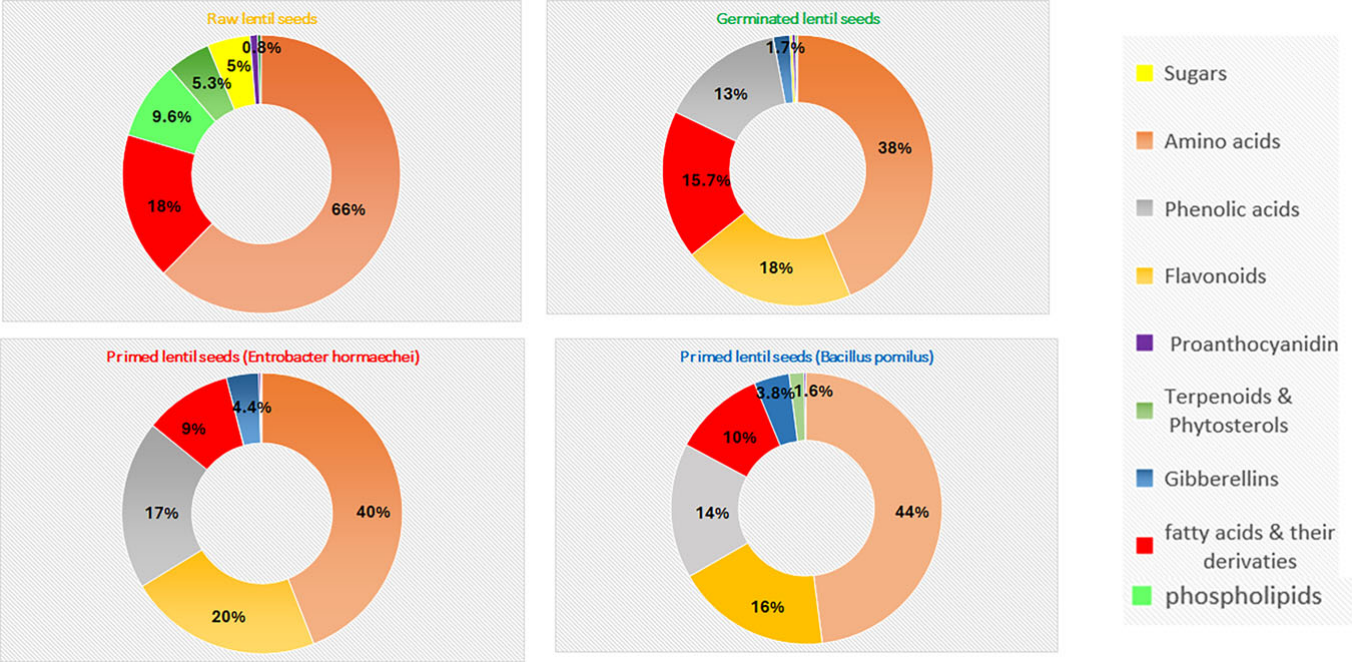
Ring diagrams readily uncovering the metabolic pattern of the main chemical classes among the different lentil samples.

**Fig. 3:**
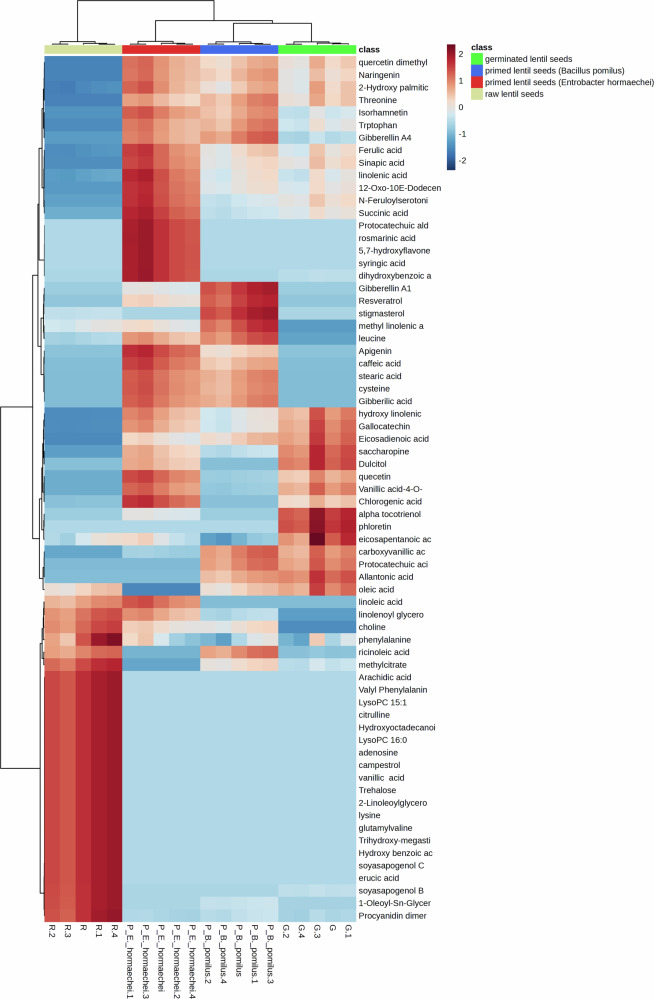
Heatmap graphically displaying the relative abundance of metabolites existing in the different lentil samples from high to low using a color-coded scale ranging from red to green.

Considering this analysis, an uneven distribution of chemical classes was observed among the different lentil samples. To facilitate visual interpretation, ring diagrams (Fig. 2) and a color-graded heatmap (Fig. 3) were generated to uncover the chemical heterogeneity across the tested samples.

As shown in the ring diagrams (Fig. 2), amino acids and sugars witnessed a striking accumulation in raw (ungerminated) seeds but were significantly reduced in germinated samples. This reduction suggests that amino acids are degraded during germination and utilized as an energy source for seedling growth^47^. Similarly, stored sugars such as trehalose in dormant seeds were rapidly mobilized and broken down at the onset of germination, providing the energy and carbon necessary for seedling development^48^.

In contrast, phenolic acids and flavonoids demonstrated a notable increase in all sprouted samples compared to raw seeds, with a slight elevation in primed samples (Fig. 2). Germination promotes the release of bound phenolics, converting insoluble forms into free (soluble) phenolics, thereby enhancing their overall content and bioaccessibility^49^. These findings are consistent with previous studies reporting that germination can increase the total phenolic and flavonoid content in chickpeas by approximately threefold compared to ungerminated seeds^50,51^.

Furthermore, biopriming can activate key enzymes such as phenylalanine ammonia-lyase (PAL), essential for the phenylpropanoid pathway, and *β*-glucosidase, which releases phenolic compounds previously bound to complex sugars in the seed^52,53^. During biopriming, seeds are coated with beneficial microorganisms that act as elicitors or biocontrol agents, triggering a cascade of plant metabolic pathways^19^.

Consistent with recent findings, biopriming of lettuce (*Lactuca sativa* L. cv. Rabicon) with *Pseudomonas putida* P10 significantly increased the contents of total phenolics, carotenoids, vitamin C, and flavonoids^54^. Further, basil (*Ocimum* L.) seeds bioprimed with *Pseudomonas* JP0825 showed elevated levels of vitamins, phenolics, and flavonoids, which were significantly correlated with the promising antioxidant and antimicrobial activities of the treated samples^55^.

On other domain, the microorganisms used in biopriming, such as plant growth-promoting rhizobacteria (PGPR), trigger the plant’s induced systemic resistance (ISR)^56^. This pre-emptive defense activation fortifies the synthesis of various secondary metabolites, primarily phenolics, which act as a broad-spectrum defense against potential pathogens and environmental stressors^56^.

Undoubtedly, the seed coat of lentils is particularly rich in procyanidins, predominantly existing as oligomers (dimers, trimers, and tetramers). However, these compounds were substantially leached out during germination, as illustrated in Figs. 2, 3.

Triterpenoids and phytosterols exhibited higher abundance in raw samples compared to treated ones. Notably, a significant increase in gibberellins was observed in germinated bioprimed samples (Fig. 3). Gibberellins naturally accumulate during seed germination to break dormancy and promote embryo growth. Biopriming further amplifies their levels by directly influencing the plant’s hormonal pathways^57^. Moreover, recent evidence indicates that beneficial microbes employed in biopriming stimulate the release of phytohormones such as gibberellins and indole-3-acetic acid, thereby activating key metabolic processes^58^.

Following germination, a moderate increase in fatty acid content was detected compared to raw seeds; however, a slight decline was noted in bioprimed germinated samples. Additionally, a pronounced reduction in phospholipids occurred during germination, as these molecules serve as a source of fatty acids that are enzymatically metabolized to meet the energy demands of the developing seedling^59^.

The observed metabolic transitions across raw, germinated, and bioprimed lentil samples reflect coordinated biochemical shifts driven by both germination and microbial stimulation. The marked reduction in sugars and free amino acids following germination indicates active mobilization of storage reserves to fuel early seedling development. In contrast, the consistent elevation of phenolic acids and flavonoids—particularly in bioprimed sprouts—suggests activation of phenylpropanoid metabolism, frequently induced by developmental and microbe-associated signaling. Amplification of these phytochemicals in bioprimed groups supports the role of plant–microbe interaction in triggering secondary metabolism and enhancing the bioactive potential of sprouts.

### Chemical discrimination of the different lentil samples using multivariate statistical analysis

To manage the complex chemical data obtained from UPLC-MS analysis, a preliminary unsupervised exploratory approach, principal component analysis (PCA), was applied to gain deep insights into hidden relationships, clustering patterns, and variability among the examined samples, presented in a simplified graphical format.

The four-component PCA model (Fig. S1) successfully segregated the lentil samples into three major clusters along the first two principal components, accounting for 73.5% of the total variance and indicating substantial differences in their chemical profiles. Specifically, raw lentil seed samples (unprocessed) exhibited a distinct chemical composition, as evidenced by their clear separation and clustering on the negative side of PC1, away from all other samples. In contrast, germinated and bioprimed lentil seeds clustered together on the positive side of PC1, suggesting a relative similarity in their chemical composition. Furthermore, PC2 effectively differentiated lentil samples primed with *E. hormaechei* from both unprimed sprouts and those bioprimed with *B. pumilus*, with the latter aggregated in the lower right quadrant along PC2.

**Fig. 4:**
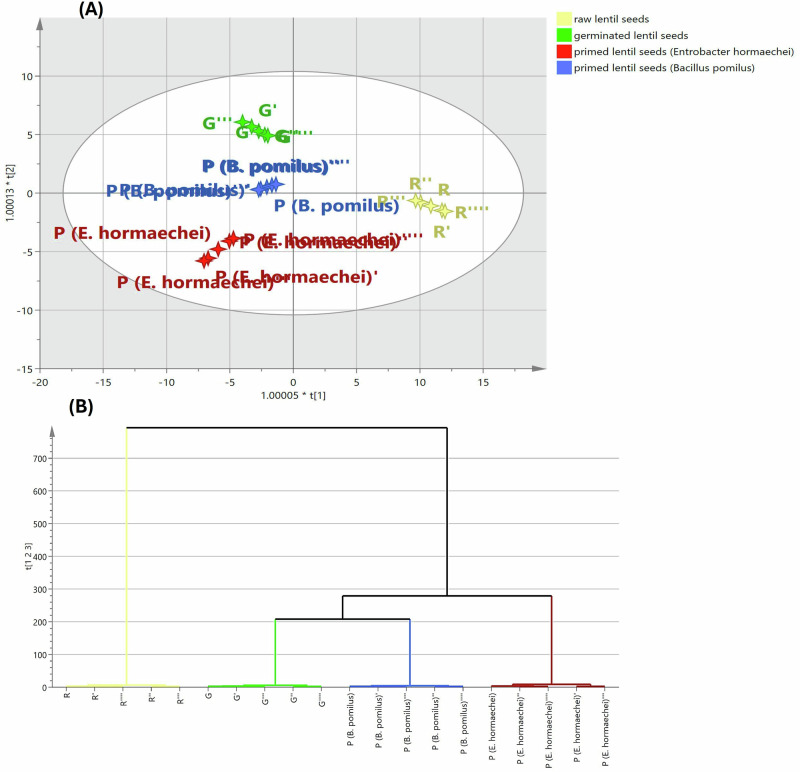
Chemical discrimination of different lentil samples (raw, germinated unprimed, and bioprimed germinated) using OPLS-DA modelling. OPLS-DA score scatter plot of different lentil samples (**A**). The respective dendrogram of hierarchical cluster analysis (HCA) (**B**).

To gain deeper insights into the metabolic alterations induced by sprouting and biopriming treatments, a supervised OPLS-DA model was constructed. The model exhibited excellent performance metrics, with *R*^2^ = 0.984 and *Q*^2^ = 0.973, confirming its strong predictive and discriminant capability. As illustrated in Fig. 4A, the OPLS-DA score plot revealed a clear separation pattern: sprouted and bioprimed samples were positioned on the opposite side of latent variable 1 (LV1) relative to raw samples, indicating statistically significant differences in chemical composition. These findings underscore the profound impact of sprouting on lentil seed chemistry, activating key metabolic processes, altering macromolecule levels, and enhancing the bioavailability of beneficial compounds. Notably, sprouts bioprimed with *E. hormaechei* were distinctly separated from unprimed sprouts along the negative direction of LV2, reflecting compositional divergence. Similarly, sprouts bioprimed with *B. pumilus* exhibited moderate deviation from unprimed sprouts, further highlighting chemical heterogeneity among treatments.

In parallel, the dendrogram of the analyzed samples revealed two distinct clusters (Fig. 4B). The first cluster comprised the raw samples, whereas the other samples were grouped together in the second cluster, indicating their relative chemical similarity. Furthermore, the second cluster was subdivided into two sub-clusters. The first one was represented by the bioprimed sprouts treated with *E. hormaechei*. While the second cluster comprised unprimed sprouts and those bioprimed with *B. pumilus*.

**Fig. 5:**
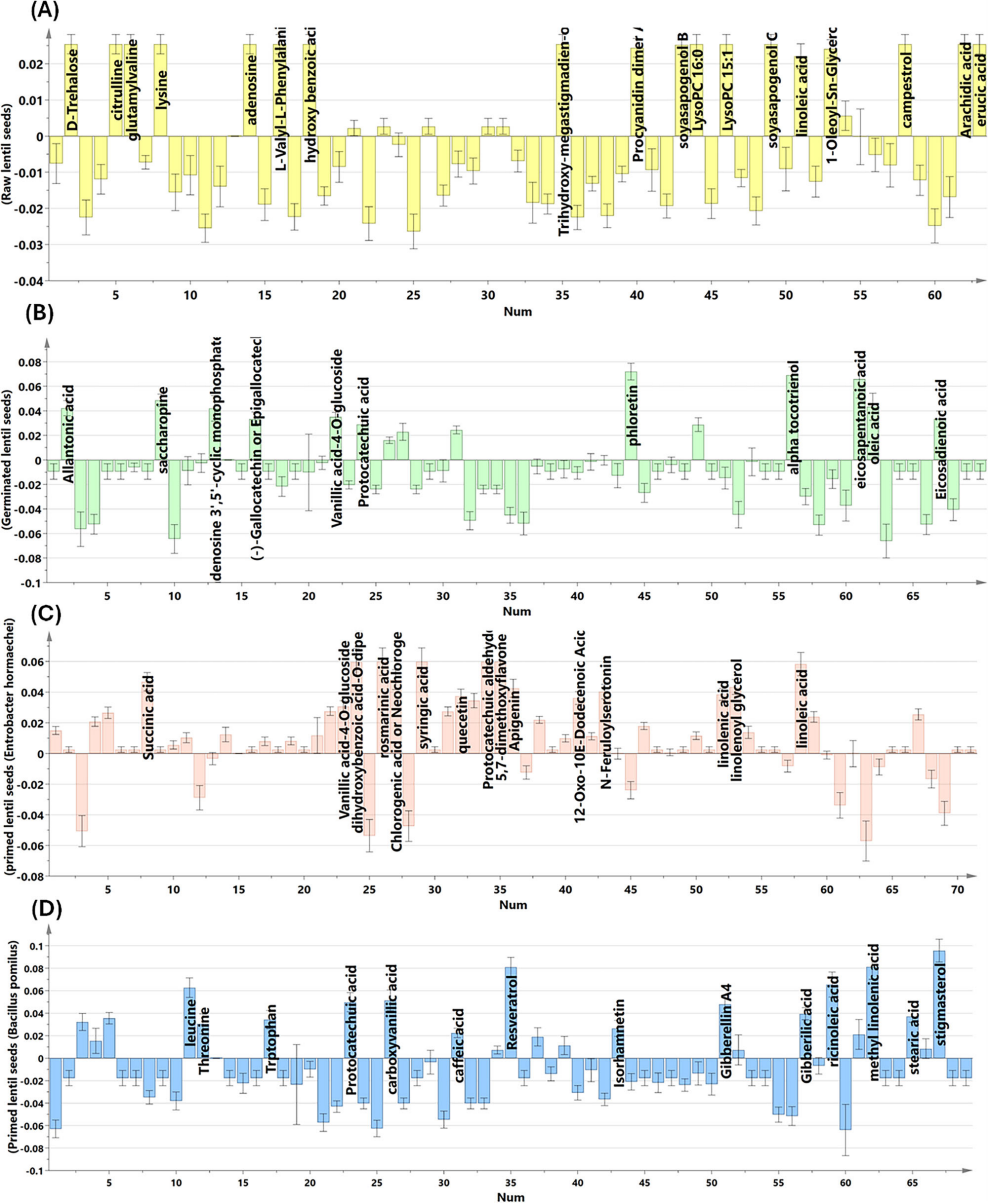
Unveiling the chemical signatures of different lentil samples (raw, germinated unprimed, and bioprimed germinated) via OPLS-DA-derived coefficient plots. Raw lentil seeds (**A**), germinated (unprimed) lentil samples (**B**), bioprimed germinated lentil samples with *E. hormaechei* (**C**), bioprimed germinated lentil samples with *B. pomilus* (**D**).

Equally important, the analysis of the OPLS-DA-derived coefficient plots (Fig. 5) identified variables (metabolites) that were highly significant for clustering and discriminating among the tested samples. Raw samples—distinguished from the other groups—were found to be enriched with trehalose, citrulline, glutamyl valine, valyl phenylalanine, trihydroxy-megastigmadien-one hexoside, procyanidin dimer A, soyasapogenol B, soyasapogenol C, LysoPC 16:0, campestrol, arachidic acid, and erucic acid, as shown in Fig. 5A. Following sprouting, certain metabolites were significantly augmented, mainly allantonic acid, gallocatechin, protocatechuic acid, phloretin, alpha tocotrienol, eicosapentanoic acid, oleic acid, and eicosadienoic acid (Fig. 5B). On the other hand, the main metabolites that differentially accumulated in sprouts bioprimed with *E. hormaechei* included vanillic acid-*O*-glucoside, dihydroxybenzoic acid-*O*-dipentoside, rosmarinic acid, syringic acid, ferulic acid, quercetin, protocatechuic aldehyde, 5,7-dihydroxyflavone, apigenin, feruloyl serotonin, linolenic acid, linolenoyl glycerol, and linoleic acid. (Fig. 5C). Sequentially, the sprouts bioprimed with *B. pomilus* witnessed a striking accumulation of protocatechuic acid, carboxyvanillic acid, resveratrol, isorhamnetin, gibberellic acid, gibberellin A_4_, leucine, cysteine, tryptophan, methyl linolenic acid, and stigmasterol (Fig. 5D).

These findings indicate substantial chemical reorganization first by germination and then by microbe-dependent remodeling. While raw samples clustered separately due to the dominance of storage molecules, germinated samples reflected de novo secondary metabolite production. Germination mobilized primary reserves and elevated phenolics and flavonoids; biopriming further accentuated these pathways in a strain-dependent manner, consistent with microbe-elicited remodeling of phenylpropanoid and hormone-associated metabolism. The unique displacement of bioprimed groups along additional discriminant vectors corroborates that bacterial inoculation induced treatment-specific chemical signatures beyond germination alone.

The validity and robustness of the OPLS-DA model in clear discrimination among the sample groups were confirmed through the 20-permutation tests (Fig. S2).

### In vitro α-amylase and α-glycosidase inhibitory activities and glucose uptake-enhancing potential of different lentil samples

**Fig. 6:**
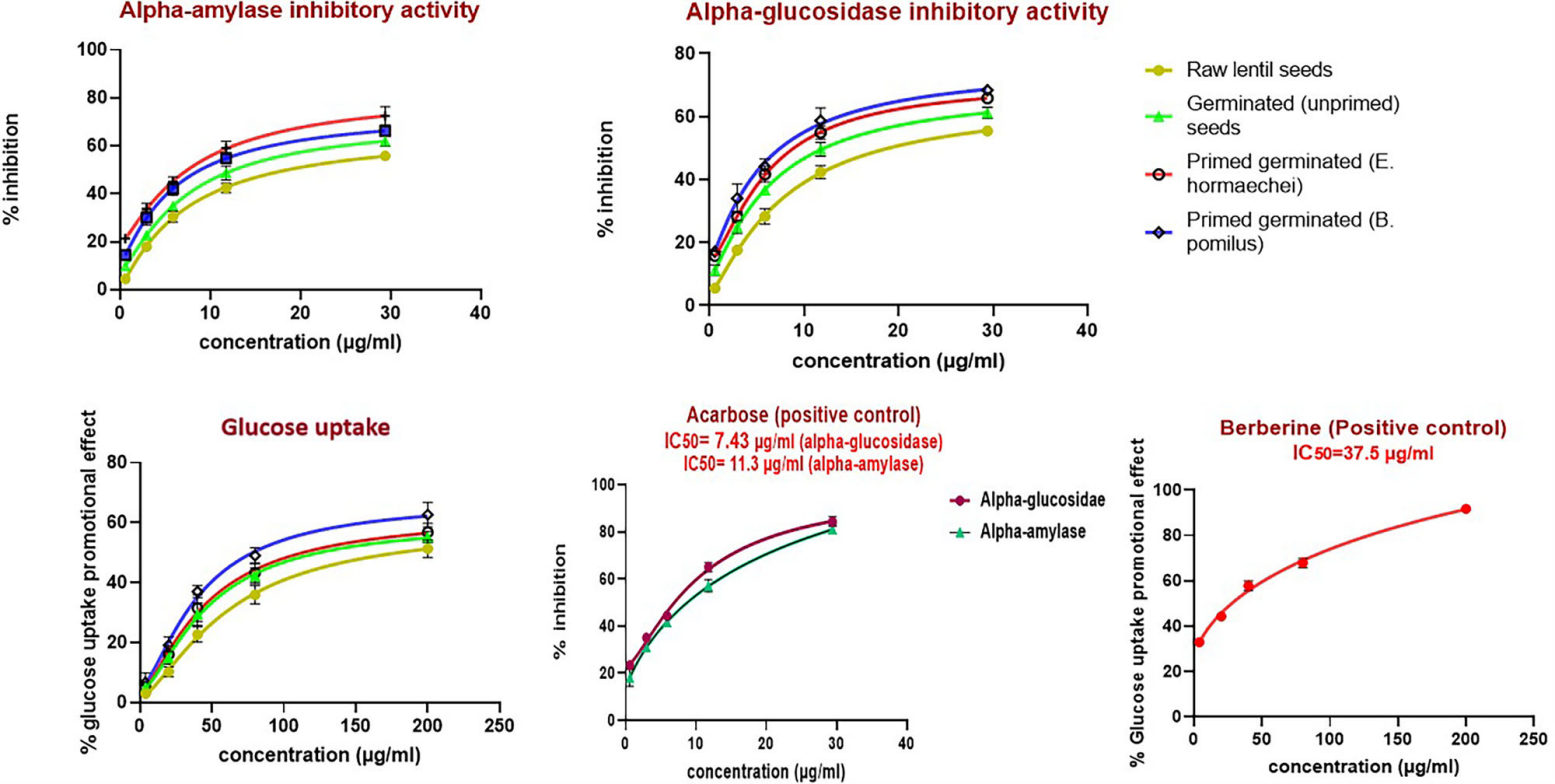
The dose–response curves of the different lentil samples displaying their inhibitory effects against *α*-amylase and *α*-glucosidase enzymes, along with the glucose uptake promotional effect.

In the subsequent experiments, different lentil samples were further screened for their *α*-amylase and *α*-glucosidase inhibitory activities, as well as their glucose uptake–promoting effects. The obtained results revealed that all lentil samples exhibited significant inhibitory activities against *α*-amylase and *α*-glucosidase enzymes, as well as a glucose uptake–promoting effect, in a dose-dependent manner. The bioprimed samples exhibited the most notable findings across all conducted assays, comparable to those of the positive control (Fig. 6). Regarding *α*-amylase and *α*-glucosidase inhibitory activities, bioprimed sprouts showed the strongest effects against both enzymes. Samples preliminarily bioprimed with *E. hormaechei* significantly inhibited *α*-amylase and *α*-glucosidase, with IC_50_ values of 8.46 ± 1.5 µg/mL and 10.19 ± 1.45 µg/mL, respectively. Similarly, bioprimed samples with *B. pumilus* demonstrated promising results, with respective IC_50_ values of 10.16 ± 0.36 µg/mL and 9.29 ± 0.466 µg/mL. Notably, unprimed sprouts also exhibited favorable inhibition compared to raw samples, with IC_50_ values of 14.4 ± 0.42 µg/mL and 13.5 ± 0.316 µg/mL, respectively.

Conjointly, the glucose uptake–enhancing potential of the extracts was evaluated by quantitatively assessing their ability to promote glucose transport across the yeast cell membrane^60^. Among the tested extracts, bioprimed sprouts with *B. pumilus* and *E. hormaechei*, as well as unprimed sprouts, significantly enhanced glucose uptake, showing IC_50_ values of 129.13 ± 0.32 µg/mL, 147.89 ± 0.466 µg/mL, and 148.69 ± 0.466 µg/mL, respectively. In contrast, raw seeds exhibited the lowest glucose absorption-enhancing effect, with an IC₅₀ of 233.46 ± 0.38 µg/mL (Fig. 6).

The enhanced α-amylase and α-glucosidase inhibition upon sprouting underscores the profound role of germination in triggering complex biochemical changes that enhance the synthesis and accumulation of bioactive compounds, thereby improving the functional attributes of edible seeds^61^. The extra Enhancement in α-amylase and α-glucosidase inhibition in bioprimed sprouts demonstrates that microbial priming strengthens functional properties beyond germination. This highlights the positive impact of biopriming on enhancing bioactive compounds production. This effect is likely attributed to the creation of a favorable environment for lentil seed germination, reconfiguration of primary and secondary metabolic pathways, and activation of metabolic and defensive cascades. These processes ultimately lead to increased synthesis of valuable secondary metabolites, such as phenolic acids, flavonoids, and omega-3 fatty acids, which possess anti-diabetic potential.

### Efficacy-driven discrimination among the different lentil samples using OPLS analysis

**Fig. 7:**
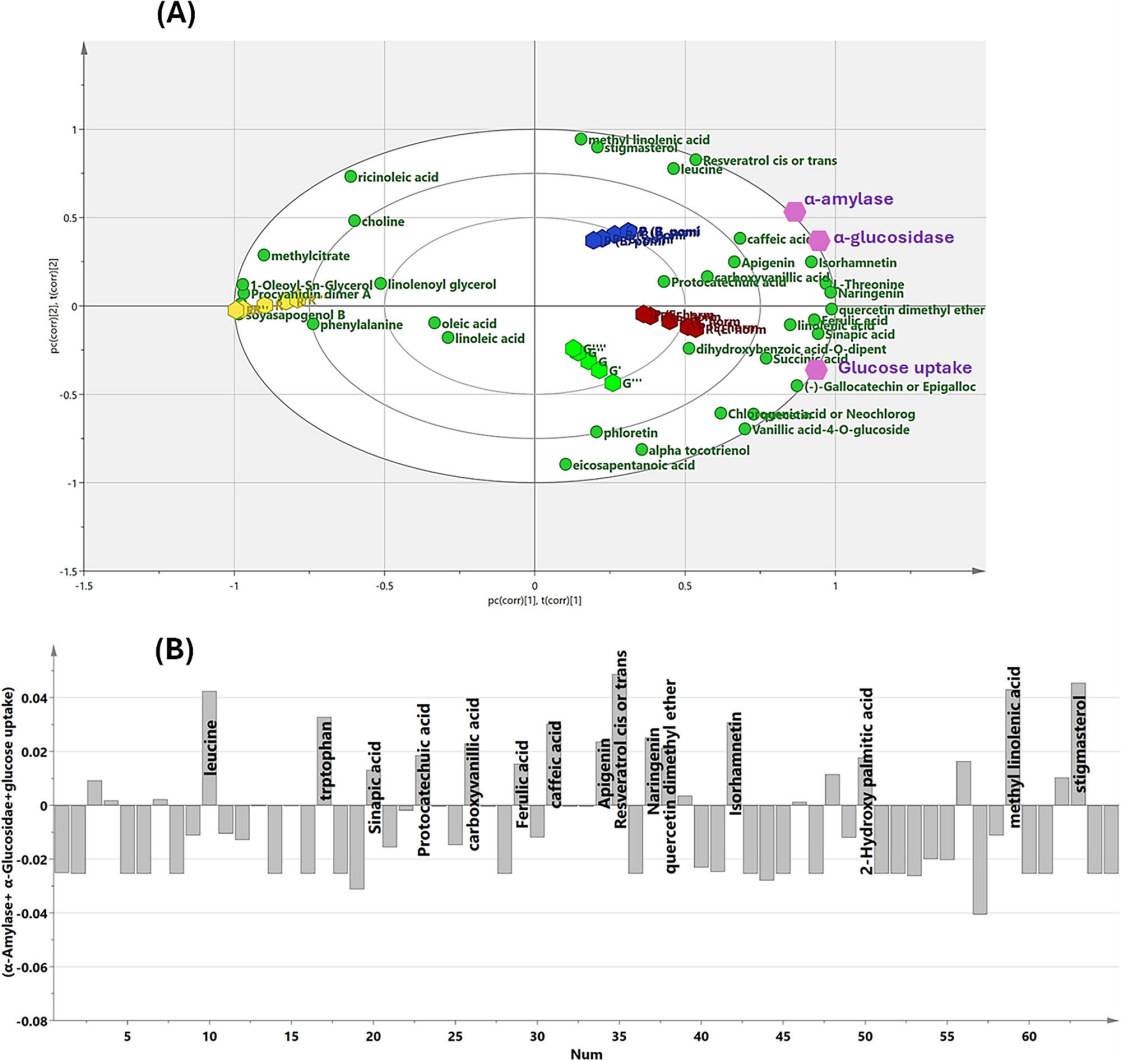
Efficacy-directed discrimination of the different lentil samples using OPLS analysis. OPLS biplot of the analysed samples (**A**), OPLS-derived coefficient plot tracing the biologically active compounds beyond α-amylase and α-glucosidase inhibitory effects as well as glucose uptake-promotional effect (**B**).

To enhance the biological interpretability of the previously analysed data, a predictive response model, orthogonal projections to latent structures (OPLS), was established to identify potential bioactive metabolites underlying the antidiabetic effect. With 74.3% of the total variation, the OPLS biplot (Fig. 7A) clearly discriminated the tested lentil samples based on their *α*-amylase and *α*-glucosidase inhibitory activities and glucose uptake–promoting effects. Germinated samples, particularly the primed ones, clustered on the positive side of latent variable 1 (LV1), showing a strong spatial correlation with the antidiabetic activity (Y-response), aligning with the previously discussed biological findings. In contrast, raw samples grouped on the opposite side, distant from *α*-amylase and *α*-glucosidase inhibitory activities and glucose uptake- promoting action, indicating their relatively low antidiabetic potential as previously verified.

To strengthen the biological relevance of the findings, OPLS-derived coefficient plots were integrated to identify effective metabolites potentially responsible for the observed antidiabetic effects in bioprimed sprouts. Examination of the OPLS-derived coefficients plot (Fig. 7B) revealed that the functional gains in glucose uptake and enzyme inhibition align with microbe-driven enrichment of key phenolic compounds, primarily sinapic acid, ferulic acid, caffeic acid, apigenin, resveratrol, naringenin, and isorhamnetin. These compounds are well recognized for their exceptional antidiabetic potential and exhibited the highest abundance in bioprimed sprouts. Additionally, dominant compounds such as leucine, tryptophan, linolenic acid, and stigmasterol may further contribute to the observed antidiabetic effects.

Consistent with previous studies, resveratrol demonstrated promising antidiabetic potential by improving glycaemic control, reducing insulin resistance, and mitigating oxidative stress^62^. Its mechanism involves activation of key cellular pathways, including AMPK and SIRT1, and induction of glucose transporter expression^63^. Similarly, our findings align with earlier reports highlighting the significant antidiabetic effects of phenolic acids such as ferulic, caffeic, and sinapic acids. These compounds act through multiple mechanisms: enhancing insulin sensitivity, inhibiting carbohydrate-digesting enzymes, modulating glucose absorption, promoting insulin secretion and pancreatic *β*-cell function, countering chronic oxidative stress associated with type 2 diabetes^64,65^.

Moreover, Recent studies have highlighted isorhamnetin as a promising antidiabetic agent, capable of reducing blood glucose levels, improving insulin sensitivity, and alleviating complications associated with diabetes, particularly Type 2^66^. Its mechanisms include reducing oxidative stress and inflammation, enhancing glucose transport via GLUT4 upregulation, and modulating key signaling pathways such as PI3K/AKT/eNOS and JAK/STAT^67^. Likewise, linolenic acid—an omega-3 polyunsaturated fatty acid—has been linked to a lower risk of Type 2 Diabetes by improving insulin sensitivity and exerting potential glucose-lowering effects^68^. Furthermore, animal studies have demonstrated that prolonged leucine supplementation improves glucose and insulin homeostasis, potentially through anti-inflammatory mechanisms^69^.

Altogether, the enhancement in the antidiabetic attributes of the bioprimed lentil sprouts correlates with the accumulation of key phenolic bioactives—well reported for carbohydrate-hydrolyzing enzymes inhibition and insulin sensitization. The convergence of elevated phenolic bioactives with superior enzymatic inhibition suggests that microbe-stimulated phenylpropanoid activity is a primary driver of antidiabetic potential. The increased glucose uptake in primed sprouts further supports the metabolic benefits imparted by microbial treatment. Lipidomic markers such as linolenic acid also contributed to cellular glucose transport enhancement. These combined mechanisms suggest that microbial priming creates a phytochemical profile more suited for glycemic regulation than germination alone.

To sum up, the profound shifts in primary and secondary metabolites, notably phenolic acids, flavonoids, and gibberellins, in bioprimed sprouts were translated into enhancement in their antidiabetic potential, evidenced by strong inhibition of carbohydrate-hydrolyzing enzymes, *α*-amylase and *α*-glucosidase, and improved glucose uptake. These findings suggest that biopriming with beneficial microbes, coupled with germination, emerges as a powerful, eco-friendly approach to reconfigure lentil seed chemistry and amplify its health-promoting attributes.

While our current investigation presented significant results, some limitations should be outlined. First, only two bacterial strains were evaluated; exploring additional strains individually or in combination may uncover complementary functional traits capable of further improving germination performance, nutrient mobilization, and secondary metabolite biosynthesis. Second, while metabolomics provided comprehensive coverage of biochemical alterations, integrating transcriptomic or proteomic analyses would enable deeper insight into regulatory gene networks and enzymatic pathways governing microbe-induced metabolic remodeling. Third, the current study relies on in vitro antidiabetic assays, which, although informative, cannot fully capture systemic metabolic responses. Thus, in vivo investigations are essential to validate the glycemic-modulating potential of bioprimed lentil sprouts and to assess any broader physiological impacts. Future work should also evaluate the bioactivity of individual metabolites implicated in the observed antidiabetic effects, quantify colonization dynamics, determine the scalability and safety of microbial inoculants during sprout production and storage, and investigate and cross-legume applicability to advance nutraceutical innovation.

## Methods

### Chemicals and reagents

Assay kits for glucose uptake, *α*-amylase, and *α*-glucosidase, along with acarbose and berberine (used as positive controls), were obtained from Sigma-Aldrich (St. Louis, MO, USA). Chromatographic-grade solvents, including methanol, acetonitrile, and formic acid for LC–MS analysis, were purchased from Merck (Darmstadt, Germany).

### Bacterial inoculum suspension preparation

*Enterobacter hormaechei* strain 3 C (GenBank accession no.: KX036556) was sourced from the Environmental Studies and Research Unit (ESRU), Department of Microbiology, Faculty of Agriculture, Cairo University. *Bacillus pumilus* (1486 T) was obtained from the Microbiological Resources Center, Faculty of Agriculture, Ain Shams University, Egypt. Each strain was cultured separately in nutrient broth (HiMedia Laboratories) at 37 °C and pH 7.4 for 48 h until reaching a concentration of 10⁸ CFU/mL. The sterilized medium contained 5 g/L peptone, 5 g/L sodium chloride, 1.5 g/L beef extract (HM peptone B), and 1.5 g/L yeast extract.

### Lentil seeds acquisition and preparation

Brown lentil seeds were procured from Cairo University’s Agricultural Research Centre (Egypt) in March 2024. Seeds of uniform size (200 g) were surface-sterilized using 0.5% sodium hypochlorite for 5 min, rinsed thoroughly, and dried in a low-temperature oven (≈38 °C) for 8 h with occasional stirring. The seeds were divided into four equal portions (50 g each). The first portion was ground, labeled as raw lentil seeds (R), and stored at 4 °C for subsequent analysis.

### Ten-day germination of lentil seeds

The second portion underwent a 10-day germination process following^61^ procedure with minor modifications. Briefly, seeds were placed on filter paper in a biochemical container and incubated in darkness at 25 ± 2 °C and 60% relative humidity. Seeds were misted twice daily. Sprouted seeds were lyophilized, powdered, labeled as germinated lentil seeds (G, unprimed), and stored at 4 °C.

### Seed priming with bacterial inoculants and subsequent germination

The remaining two portions were independently subjected to three priming treatments using *E. hormaechei* and *B. pumilus* prior to germination. Bacterial suspensions were prepared by culturing each strain in Luria–Bertani (LB) medium at 37 °C (180 rpm) until reaching 10⁸ CFU/mL, then diluted to 10⁵ CFU/mL as the optimal concentration for plant growth^24,70^. Sterilized seeds were separately submerged in bacterial suspensions for 8 h to allow colonization and initiate pre-germination metabolism. After priming, seeds were germinated as described above, lyophilized, ground, and labeled as (P, *E. hormaechei*) and (P, *B. pumilus*). All samples were stored at 4 °C.

### Extraction of different lentil samples

Following previously optimized protocols^71,72^, 50 g of each sample were extracted twice with 200 mL of 70% ethanol at 40 °C for 60 min using an ultrasonic bath (3 L Alpha Plus, Japan). Extracts were concentrated to dryness using a rotary evaporator (Buchi Rotavapor, Switzerland). Five replicates of each sample were prepared for subsequent analyses.

### Phytochemical profiling of different lentil extracts via UPLC-MS/MS

Phytochemical profiling was performed using an XEVO UPLC triple quadrupole (QqQ) system coupled with an electrospray ionization (ESI) source (Waters Corporation, Milford, MA, USA). Chromatographic conditions, ESI parameters, and data processing details followed^72,73^ protocols and are provided in the Supplementary Material.

### Screening of antidiabetic potential of the different lentil seed/sprout extracts using α-amylase and α-glucosidase inhibitory activities along with glucose uptake assays

The inhibitory activity of *α*-amylase (EC 3.2.1.1) was assessed following the method described by ref. ^74^ with minor modifications. Briefly, 10 µL of each tested extract at varying concentrations (10, 50, 100, 200, and 500 µg/mL) was mixed with 50 µL of *α*-amylase solution (5 mg/0.5 mL in 0.1 M phosphate buffer, pH 6.9) in a 96-well plate. After the addition of 50 µL of 1% (w/v) dextrin substrate solution, the plate was incubated for 45 min. Subsequently, 100 µL of glucose kit reagents (AAP, 1 mM/L; GOD > 20 KU/L; NaN₃, 8 mmol/L) were added, and absorbance was measured at 580 nm. Acarbose served as the positive control, while sodium phosphate buffer (pH 7.4) was used as the negative control. The *α*-amylase inhibitory activity of the tested samples was expressed as IC₅₀ (µg/mL), calculated as the mean ± SD (*n* = 3).

The *α*-glucosidase (EC 3.2.1.20) inhibitory activity was evaluated following the method described by ref. ^74^ with minor modifications. Briefly, an *α*-glucosidase solution was prepared in potassium phosphate buffer (pH 7.4) and mixed with 10 µL of each sample at varying concentrations (10, 50, 100, 200, and 500 µg/mL). The reaction mixture was supplemented with an equal volume of bovine pancreatin enzyme solution (5 mg/0.5 mL in 0.1 M phosphate buffer, pH 7.4) and incubated at 35 °C for 20 min. Subsequently, 50 µL of *p*-nitrophenyl-*α*-D-glucopyranoside (PNPG, 5 mM) was added, and the reaction continued for an additional 15 min. The reaction was terminated by adding 1 M Na_2_CO_3_, and the absorbance was measured at 400 nm using a spectrophotometer. The *α*-glucosidase inhibitory activity of the tested samples was expressed as IC₅₀ (µg/mL), calculated as the mean ± SD (*n* = *3*).

Following a previously reported protocol^75^ with minor modifications, a glucose uptake assay was performed. In this assay, baker’s yeast absorbs glucose via glucose transporters in the presence of plant extracts, after which the glucose is metabolized into glucose-6-phosphate. The residual glucose is then quantified using glucose oxidase and peroxidase kit reagents. Baker’s yeast was centrifuged (3000 × *g*, 5 min) with distilled water until the supernatant became clear, and a 10% (v/v) suspension was prepared. Subsequently, 1 mL of seed extracts at varying concentrations (10, 50, 100, 200, and 500 µg/mL) was mixed with 1 mL of glucose solution (25 mM in distilled water) and incubated at 37 °C for 10 min. After adding 100 µL of the yeast suspension, the mixture was further incubated for 60 min at 37 °C. Each tube was then centrifuged (2500 × *g*, 5 min). The remaining glucose in both sample and control tubes was determined using glucose oxidase and peroxidase kit reagents, with phosphate buffer supplied in the blank tube. Berberine served as the positive control. The glucose uptake-enhancing potential of the seed extracts was expressed as IC₅₀, defined as the concentration required to promote 50% of the glucose uptake process.

### Statistical analysis

Data were expressed as means ± standard deviations (SD). Statistical analyses were performed using GraphPad Prism v8 (GraphPad Software, San Diego, CA, USA). Comparisons between two groups were conducted using Student’s *T*-test, while comparisons among more than two groups were assessed using one-way analysis of variance (ANOVA). A *p* < 0.05 was considered statistically significant. For chemometric analysis^76^, UPLC-MS data were processed using SIMCA-P software (version 14.1; Umetrics, Umea, Sweden). Principal Component Analysis (PCA) was initially applied as an exploratory tool to visualize clustering patterns and detect outliers within the high-dimensional dataset. Subsequently, Orthogonal Projections to Latent Structures-Discriminant Analysis (OPLS-DA) was employed as a supervised classification method to optimize sample separation and identify key chemical features distinguishing lentil samples, thereby improving model robustness through coefficient plots. Additionally, an Orthogonal Projections to Latent Structures (OPLS) model was constructed as a biologically predictive tool to identify major bioactive compounds potentially responsible for the observed glucose uptake-promoting effect and *α*-amylase and *α*-glucosidase inhibitory activities of lentil extracts. Model performance, goodness-of-fit, and predictive capability were evaluated using two statistical parameters: *R*^2^ (explained variance) and *Q*^2^ (predictive variance). Permutation tests (*n* = 20) were conducted to assess potential overfitting and ensure the robustness and reliability of the supervised models.

## Supplementary information

Supplementary Information

## Data Availability

The datasets generated and/or analysed during the current study are not publicly available due to the proprietary nature of the specialized tools and computational models used, but are available from the corresponding author on reasonable request.
