# Supplementary material for: Microbial biopriming and germination cooperatively remodel brown lentil seeds (Lens culinaris L.) metabolome and antidiabetic functionality
Source: NPJ Sci Food. 2026 Apr 13;10:160. doi: 10.1038/s41538-026-00824-5 (PMC13194844; doi:10.1038/s41538-026-00824-5)
Supplement: Supplementary file 1 — Supplementary Information [file 41538_2026_824_MOESM1_ESM.pdf]

UPLC experiment conditions

Comprehensive metabolomics analysis of lentil extracts was achieved using ultrahigh-performance liquid chromatography coupled with triple quadrupole tandem mass spectrometry (UHPLC-QqQ-MS/MS) through electrospray ionization-mass spectrometry (ESI-MS) positive and negative ion acquisition mode carried out on a XEVO QqQ triple quadruple instrument (Waters Corporation, Milford, MA, USA). The UPLC system encompassed a Waters Acquity QSM pump, a LC-2040 (Waters Corporation) autosampler, degasser and Waters Acquity CM detector. Samples were separated using a Waters Acquity UPLC BEH C18 column (1.7 µm particle size – 2.1 × 50 mm). A binary mobile phase was prepared by filteration using 0.2 µm filter comprising membrane disc and degassed by sonication before injection. The mobile phase consisted of water + 0.1% (v/v) formic acid (A) and methanol + 0.1% (v/v) formic acid (B). The mobile phase was pumped at 0.2 mL/min into the UPLC system with injection volume of 5 µL and programmed as gradient elution through 32 min performed as the following sequences: 0.0–2.0 min, 10% B; 2.0–5.0 min, 30% B; 5.0–15.0 min, 70% B; 15.0–22.0 min, phase 90% B; 22.0– 25.0 min, 90% B; 26.0 min, 100% B; 26.0–29.0 min, 100% B; 29.0–30.0 min, 10% B.

ESI-MS conditions

For LC/MS analysis, a triple quadruple (QqQ) mass spectrometer was coupled to the UPLC instrument via an ESI interface. Ultra-high purity helium (He) was used as the collision gas and high purity nitrogen (N_2) as the nebulizing gas. The mass spectrometer was monitored in both negative and positive ionization modes over 50–1200 *m/z* mass range. The optimized detection parameters were as follows: temperature 150 °C, cone voltage 30 V, capillary voltage 3 kV, desolvation temperature 440 °C, cone gas flow 50 L/h, and desolvation gas flow 900 L/h. The analysis process run time lasted for 32 min. Regarding automatic MS/MS fragmentation process of the precursor ions that have been filtered by the first quadrupole (Q1), then in the second quadrupole (Q2) the mass fragmentation was performed through collision-induced dissociation (CID) energy that was ramped from 30 to 70 eV utilizing Ultra-high purity helium as collision gas. Eventually, the third quadrupole mass analyzer (Q3) filtered the daughter ions produced from CID that consequently related to the molecular structure of the precursor ions.

Annotation of UPLC-MS/MS metabolites

The raw UPLC–MS data were pre-processed using Mzmine® version 2.8 software that has been utilized for importing data, peak deconvolution, alignment, and annotation.

Tentative assignment of metabolites was established via comparing their retention times relative to external standards, interpreting tandem mass spectra (quasi-molecular ions as well as diagnostic MS/MS fragmentation profiles) combined with our in-house comprehensive database that was set up covering all compounds previously reported in the literature in lentil including Dictionary of Natural Products (https://dnp.chemnetbase.com/), Puchem and Massbank (https://massbank.eu/MassBank/) to provide high confidence level of annotation.

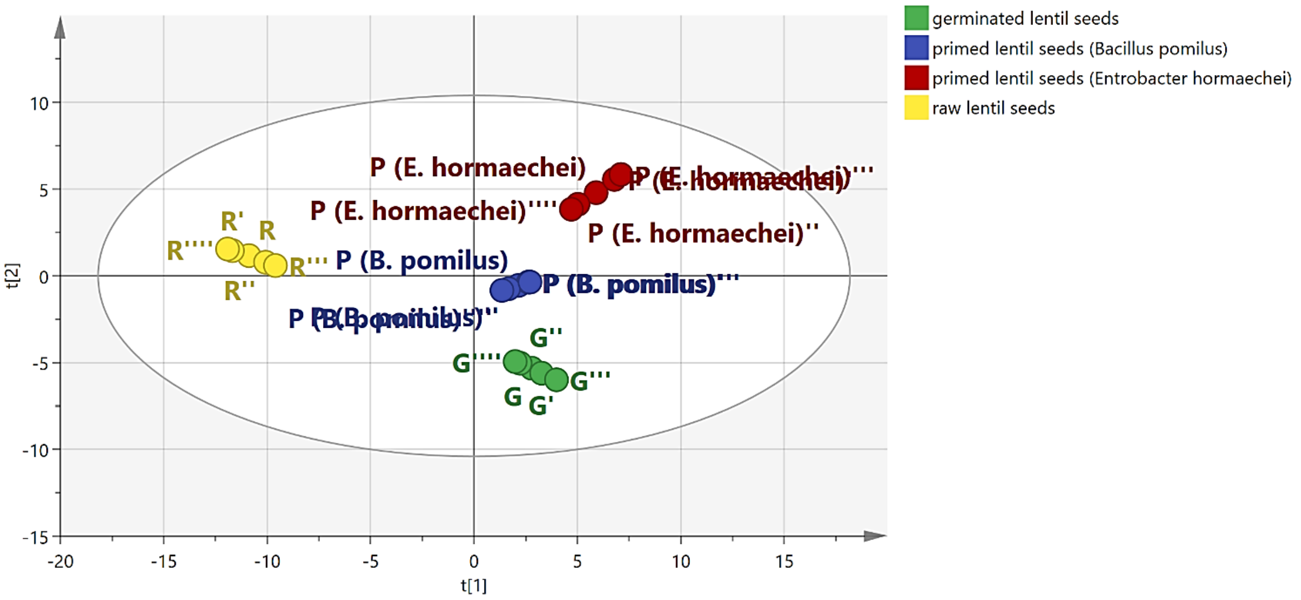

Figure S1: PCA score plot of different lentil extracts

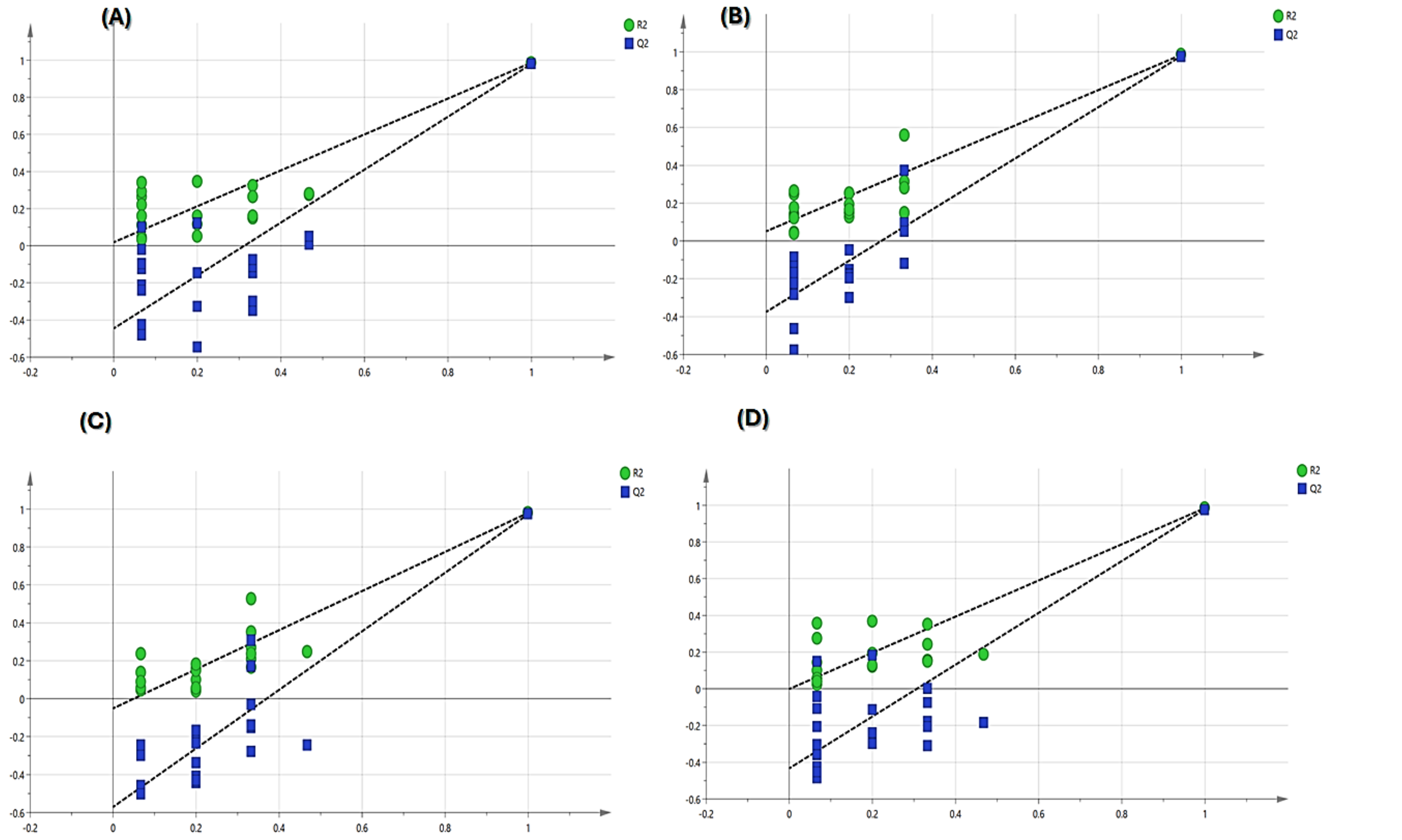

Figure S2: Permutation tests of different lentil extracts**.** Raw lentil seeds (**A**) germinated (unprimed) lentil samples (**B**), Bioprimed germinated lentil samples with *E. hormaechei* **(C).** Bioprimed germinated lentil samples with *B. pomilus* **(D).**
